# The Colombian Medical Cannabis Paradox: A Scoping Review of Structural Barriers and Health Inequity

**DOI:** 10.3390/ijerph22121792

**Published:** 2025-11-26

**Authors:** Olga Patricia Marín Arroyave, Pedro León Cruz

**Affiliations:** 1 Grupo de Investigación en Salud Integral (GISI), Departamento Facultad de Salud, Universidad Santiago de Cali, Cali 760035, Colombia; decanaturasalud@usc.edu.co; 2 Grupo de Investigación en Sostenibilidad Empresarial, Social y Ambiental (GISESA), Facultad de Ciencias Económicas y Empresariales, Universidad Santiago de Cali, Cali 760035, Colombia

**Keywords:** medical marijuana, health equity, social determinants of health, legislation, drug, health services accessibility, socioeconomic factors, Colombia

## Abstract

**Background:** Law 1787 of 2016 established Colombia’s medical cannabis framework, intended as a public policy to improve health equity by guaranteeing access and promoting inclusive development. This scoping review analyzes this policy’s implementation as a social determinant of health, mapping the literature on the gap between its legislative promise and its real-world impact. **Methods:** A scoping review was conducted following PRISMA-ScR guidelines across six electronic databases, selecting peer-reviewed articles (2016–2025) that analyzed the regulatory, socioeconomic, and healthcare factors acting as structural determinants in Colombia. **Results:** From 122 initial records, 10 studies were included. The analysis reveals a systemic paradox: the policy’s implementation created structural barriers that undermine its equity goals. Findings highlight three gaps: (1) Inequitable Access: High costs and lack of medical knowledge create socioeconomic, rather than clinical, barriers for patients. (2) Socioeconomic Marginalization: The market model favors corporate capture, systematically excluding small-scale rural producers. (3) Policy-Driven Inequity: The regulatory framework’s complexity reinforces these inequities. **Conclusions:** Colombia’s medical cannabis policy implementation functions as a social determinant that produces health inequity. To align with public health objectives, policy reform must dismantle these identified structural barriers.

## 1. Introduction

Public policies are potent social determinants of health, with the capacity to either mitigate or exacerbate existing inequities. The regulation of medical cannabis in Colombia, established by Law 1787 of 2016, emerged as one of these transformative policies. It marked a paradigm shift in national drug policy and positioned the country as a potential leader in a burgeoning global industry. Its legislative framework was built on a dual promise oriented toward health equity: on the one hand, to provide patients with safe and informed access to cannabis-based therapies, and on the other, to foster an inclusive economic model that would integrate small-scale rural growers historically affected by illicit economies and armed conflict [1,2,3]. This ambitious vision sought to align public health objectives with the promotion of social equity. This approach contrasts significantly with other regulatory models. For instance, in many U.S. states and Canada, the regulatory pathway often focused primarily on patient access and market dynamics before addressing recreational use [4,5]. Conversely, Uruguay adopted a simultaneous regulation model for both uses. The Colombian framework, however, is distinct in its foundational, explicit mandate for social inclusion and rural development, underscoring the necessity of examining its implementation through the lens of health equity [6,7].

However, the transition from legislative promise to practical outcomes has revealed significant implementation gaps, hindering its equity goals. This challenge is intensified when contrasting Colombia’s strict regulatory approach, oriented toward quality and safety, with more flexible international models or with those that have achieved faster implementation of the recreational component. Global experience demonstrates that the implementation of regulatory frameworks, even those with good intentions, encounters a series of systemic facilitators and barriers that determine their success or failure, making this analysis imperative in the Colombian context [7,8]. For example, studies in Europe and Australia have highlighted persistent barriers, including a lack of medical knowledge, high product costs, and regulatory complexity, which significantly limit patient access despite legalization. The scientific evidence documents two critical facets of this disconnect, which function as structural barriers. First, in the socioeconomic sphere, a “normative asymmetry” has been consolidated, where regulation primarily benefits the corporate sector, creating formidable barriers that marginalize the small-scale producers the law intended to protect, thereby perpetuating socioeconomic inequities [9]. Second, within the health system, systemic obstacles persist, such as a lack of training and knowledge among physicians, which limits patients’ real access to quality-assured products [10,11].

The central mission of evidence-based health policies is to ensure that regulatory frameworks translate into tangible benefits for the population. In the Colombian context, evidence on the impact of cannabis regulation is fragmented across public health, rural sociology, and political science, making it difficult to achieve a comprehensive understanding of how the policy, as a whole, is functioning as a determinant of health. Understanding the documented discrepancies between intended goals and actual outcomes is, therefore, essential for identifying these structural flaws and informing future policy adjustments [2].

Consequently, this scoping review aims to systematically map the scientific evidence on the implementation of the medical cannabis framework in Colombia through the lens of health equity. It synthesizes the available literature on socio-regulatory barriers, inequities in rural development, and obstacles to patient access. By providing a comprehensive overview of this fragmented evidence, we seek to analyze the key dimensions of the implementation paradox, framing its failures not as isolated incidents but as the result of structural barriers that must be addressed to create more effective and equitable public policies.

## 2. Materials and Methods

### 2.1. Protocol and Registration

This scoping review was conducted in accordance with the methodological guidelines of the Joanna Briggs Institute (JBI) and is reported following the PRISMA-ScR (Preferred Reporting Items for Systematic reviews and Meta-Analyses extension for Scoping Reviews) framework [12,13,14,15]. The protocol was developed internally by the authors with the objective of systematically mapping the scientific literature on the implementation of Colombia’s medical cannabis framework, conceptualizing it as a social determinant of health and analyzing its impact on health equity. In line with recommendations for scoping reviews, prior registration of the protocol in a database such as PROSPERO was not performed, as this step, while good practice, is not considered mandatory for this type of review.

### 2.2. Research Question and PCC (Population, Concept, Context) Framework

To guide this review, the following question was formulated: How does the scientific literature characterize the implementation of Colombia’s medical cannabis regulation as a social determinant of health, and what structural barriers and health inequities have been documented in its outcomes? The PCC framework was employed to structure this question and guide the search strategy:

**Population (P):** Stakeholders in the medical cannabis ecosystem in Colombia, including small and medium-scale producers (farmers), companies, healthcare personnel, and patients.

**Concept (C):** The central concept is the role of the regulatory framework as a social determinant of health. This concept was operationalized by analyzing specific conceptual dimensions and indicators documented in the literature, including: structural barriers (e.g., regulatory complexity, market dynamics, bureaucratic hurdles); socioeconomic dimensions (e.g., market capture, rural marginalization, impact on small-scale producers); and health access dimensions (e.g., unequal patient access, barriers related to cost, lack of medical knowledge, and geographic availability).

**Context (C):** Peer-reviewed scientific research published since the enactment of Law 1787 in 2016, focusing on the Colombian setting.

### 2.3. Eligibility Criteria

Strict inclusion and exclusion criteria were established for study selection:

**Inclusion Criteria:** Peer-reviewed scientific articles analyzing the regulatory framework, access barriers, socioeconomic impact, or the role of small-scale producers in the medical cannabis industry in Colombia post-2016, published in Spanish or English, were included.

**Exclusion Criteria:** Theses, dissertations, editorials, opinion articles, and grey literature without explicit methodological rigor; studies focused exclusively on recreational use; and purely agronomic or pharmacological research without an explicit connection to the socio-regulatory context were excluded.

### 2.4. Search Strategy

A systematic search was conducted between January 2016 and May 2025 in six electronic databases: Scopus, Web of Science, PubMed, SciELO, Redalyc, and Google Scholar. The search was limited to articles published in Spanish or English.

The main search algorithm in Spanish was: (“cannabis medicinal” OR “marihuana medicinal”) AND (“Colombia”) AND (“marco regulatorio” OR “legislación” OR “barreras de acceso” OR “pequeños productores” OR “desarrollo rural” OR “impacto socioeconómico”).

An analogous strategy was used for the search in English: (“medical cannabis” OR “medical marijuana”) AND (“Colombia”) AND (“regulatory framework” OR “legislation” OR “access barriers” OR “small-scale producers” OR “rural development” OR “socioeconomic impact” OR “health equity” OR “access to healthcare”).

Terms within each concept were combined using the Boolean operator ‘OR’, and the concepts were combined using ‘AND’. No truncation rules were applied to maintain specificity.

### 2.5. Study Selection and Data Extraction

The identified records were managed in Zotero (v. 6.0; https://www.zotero.org/ accessed in 15 May 2025) to remove duplicates and were subsequently uploaded to Rayyan AI for the screening process. Two independent reviewers (O.P.M.A. and P.L.C.) examined the titles, abstracts, and full texts to determine eligibility, resolving discrepancies by consensus. A data extraction form was developed to chart key variables, including study type, methodology, stakeholders analyzed, identified barriers (regulatory, financial, knowledge-based, social), impact on small-scale producers, documented health access issues, and the central gap or paradox reported.

### 2.6. Synthesis and Presentation of Results

The extracted data were synthesized narratively. A thematic synthesis was performed using a combined deductive and inductive approach. A deductive framework based on the review’s primary concepts (health access barriers and socioeconomic/rural impact) was used to initially categorize the data. Subsequently, inductive coding was applied to identify specific emergent themes and structural barriers within these broader categories. The findings were then grouped thematically around the main implementation gaps identified [16]. Although formal methodological quality appraisal was not conducted, which is an acceptable practice for scoping reviews, the exclusive inclusion of peer-reviewed articles ensured a baseline of academic rigor. Methodological characteristics were noted during data extraction to contextualize the evidence (see Table 1). The study selection process is presented visually using a PRISMA flow diagram.

### 2.7. Figure Creation

The visualizations and diagrams presented in this article were generated using the Matplotlib library in Python (https://colab.research.google.com/, 3.11.5; accessed in 15 May 2025) through the Google Colab development environment. The PRISMA flow diagram was created with the online PRISMA2020 R package tool [22].

## 3. Results

### 3.1. Studies Identified for Review

**Figure 1:**
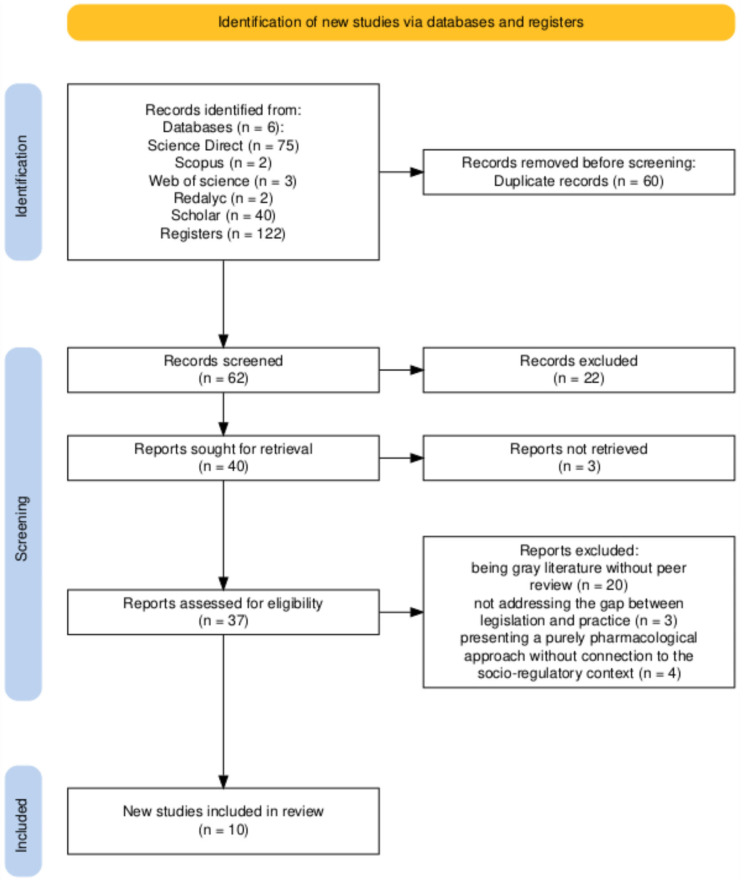
PRISMA 2020 flow diagram of study selection process. The term ‘registers’ (*n* = 122) refers to the total number of records identified through the database search. Source: own elaboration. Source: own elaboration.

The study selection process is detailed in the PRISMA flow diagram (Figure 1). The initial systematic search in the six databases specified in the methodology and reference review identified a total of 122 records (termed ‘registers’ in the PRISMA diagram, referring to all items identified by the search). After removing 60 duplicates, 62 titles and abstracts were screened. Three were removed due to a lack of free access to the content, leaving 37 manuscripts. The 37 articles were assessed in their entirety for eligibility. During this comprehensive review, 27 studies were excluded. The main reasons for exclusion were: being grey literature without peer review (*n* = 20), such as undergraduate theses; not analyzing the gap between legislation and practice (*n* = 3); and having a purely pharmacological focus with no connection to the socio-regulatory context (*n* = 4). Thus, the final number of studies included for the qualitative synthesis was 10.

### 3.2. Overview of Scientific Research: Multidisciplinary and Critical Approaches

Table 1 presents the general characteristics of the 10 studies included in the qualitative synthesis. Unlike grey literature (theses, unpublished reports), the peer-reviewed scientific corpus offers a panoramic view of the medical cannabis industry in Colombia characterized by a multidisciplinary approach and a predominantly critical perspective.

**Figure 2:**
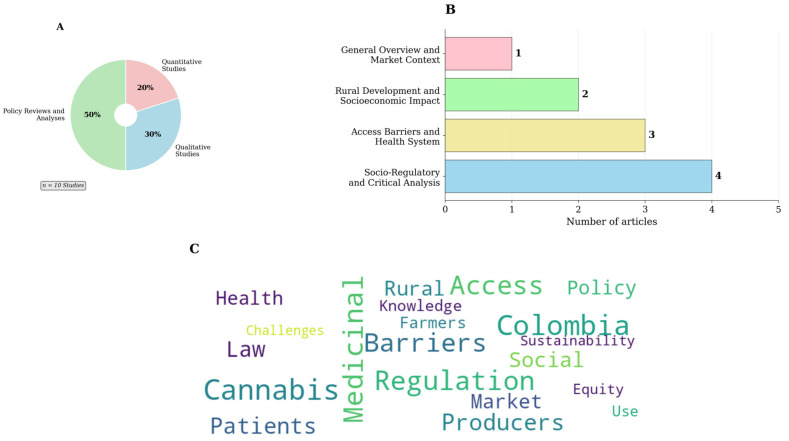
A descriptive synthesis of the 10 included studies. This figure presents a visual synthesis of the analyzed scientific research corpus (*n* = 10). (**A**) The pie chart illustrates the methodological distribution of the studies. (**B**) The bar chart quantifies the primary thematic focus of the articles, revealing a strong orientation towards socio-regulatory analysis and access barriers. (**C**) The word cloud, generated from the titles and abstracts of the 10 studies, highlights the most frequent key concepts, such as “barriers”, “regulation”, “producers”, “access”, and “patients”. The figure is intended to be descriptive and provide a clear overview of the evidence base. Source: own elaboration.

Figure 2 reveals that the landscape of scientific research on medical cannabis in Colombia is dominated by a rigorous analysis of public policy rather than economic feasibility studies or business plans. The methodological distribution (Figure 2A) shows a balanced approach, where half of the production consists of policy analyses and reviews (*n* = 5 studies; 50%), and the other half is empirical research, both qualitative (*n* = 3; 30%) and quantitative (*n* = 2; 20%). This diversity of approaches converges on a clear thematic interest (Figure 2B): the priority is socio-regulatory and critical analysis, which represents the most studied area. Together with research on access barriers and the health system, these topics shape a body of evidence focused on structural challenges and the gaps between the law’s objectives and its practical outcomes. This focus is crystallized in the word cloud (Figure 2C), which underscores the recurrence of terms like “barriers”, “regulation”, and “access”. Therefore, this core of critical and empirical research provides a robust context for the tensions in the regulatory framework and socioeconomic challenges, thus forming the basis for the analysis of the “sustainability paradox” addressed in this work.

### 3.3. Gap 1: Access to Health—Between Right on Paper and Patient Reality

The selected scientific literature consistently documents a fundamental gap between the right to access medical cannabis, guaranteed by Law 1787, and the actual ability of patients to obtain treatments in a safe, informed, and affordable manner [10,18]. Although the regulatory framework exists, its materialization is hindered by a set of systemic barriers operating at the level of healthcare personnel, costs and procedures for patients, and the social stigma that still persists.

One of the most robust findings is the profound lack of knowledge and training among medical personnel, which constitutes a primary obstacle to proper prescription and follow-up [10]. This barrier is not merely anecdotal; it is empirically confirmed in a survey study that revealed an alarming 66.2% of Colombian psychiatrists do not know how to help their patients legally access medical cannabis, and only 25% understand the current state of legislation in the country [11]. This lack of preparation generates systemic mistrust and limits the willingness to prescribe, a challenge exacerbated by the complexity of dosing and prescribing compounded preparations, which, being the main access route, require a deep knowledge of cannabinoid pharmacology that is often lacking [19]. This disconnect is aggravated by a notable bias in therapeutic acceptance: while there is majority support for use in conditions like chronic pain (87.6%), there is widespread disapproval for its use in mental illnesses. This deficiency in medical knowledge is identified as a primary implementation barrier [23,24].

In parallel to this health gap, the economic factor is identified as a decisive and, in many cases, insurmountable individual barrier for the patient [10]. The main route of access to treatments is the aforementioned compounded preparations, which, lacking sanitary registration and being individually formulated, have high costs that are not consistently covered by the health system, forcing patients to incur high out-of-pocket expenses [18]. This creates profound inequity, where effective access to treatment depends directly on the individual’s purchasing power. The lack of availability of medicines and phytotherapeutics with sanitary registration and broad coverage, coupled with the high cost of legal options, fosters a dangerous parallel market. Consequently, patients resort to products of dubious origin, artisanally made and without quality certifications, which poses a risk to their health and undermines the primary goal of “safe access” of the law [18]. This scarcity of affordable legal supply pushing patients to the informal market mirrors the paradox found in Uruguay, where a quality-oriented but excessively strict regulatory design resulted in a limited and expensive legal market [6]. Furthermore, as a factor that permeates the entire ecosystem, the literature points out that myths and negative moral connotations associated with marijuana persist both in the general population and among health professionals, acting as a social barrier that perpetuates ignorance and mistrust towards a legal therapeutic option [10].

### 3.4. Gap 2: Rural Development—Between Promised Inclusion and Documented Marginalization

The second dimension of the paradox of Colombian regulation manifests in the socioeconomic sphere, where the legislative promise of inclusion and strengthening of small-scale producers clashes head-on with a reality of marginalization and corporate capture. Law 1787 was designed with an explicit social component to protect and integrate farmers into the legal value chain, presenting itself as an unprecedented opportunity for rural development and the substitution of illicit crops [9]. However, a critical analysis of the literature suggests that, in practice, the model has not only failed to fulfill this promise but has also perpetuated historical inequities.

The concept of “normative asymmetry” is central to understanding this phenomenon, where licensing requirements, characterized by high costs, technical complexity, demanding security protocols, and burdensome bureaucracy, create insurmountable entry barriers for small and medium-sized producers [9,20]. This regulatory design, in practice, favors large companies with financial muscle and technical capacity, often with foreign capital. This exclusion is reinforced by the geographical concentration of licenses in the country’s most developed departments, such as Cundinamarca and Antioquia, far from post-conflict zones like Cauca, which the law intended to primarily benefit [9]. This demonstrates that the regulatory design, in practice, has not mitigated the systematic exclusion of small actors [25].

This perception of inequity is corroborated by empirical studies that, through surveys of growers, producers, and marketers, confirm the difficulties they face in achieving integrated and equitable rural development under the current framework [17]. Instead of transforming rural economies, the model has tended to marginalize peasant livelihoods, contrasting a legal, corporate, and high-tech market with the practices and knowledge of traditional growers, who are ironically still persecuted for cultivating the same plant [9].

### 3.5. Gap 3: The Regulatory Framework—Between Theoretical Vanguard and Practical Complexity

To understand the roots of these dysfunctions in health and rural development, it is necessary to analyze the regulatory framework itself, whose implementation has revealed a profound tension between its theoretical vanguardism and its practical complexity. The literature on the law’s formulation process suggests that its approval was an act of political cunning, achieved through “strategic uses of morality” [2]. That is, arguments with high emotional impact, such as compassion for children with epilepsy, were prioritized to overcome political opposition and the lack of consolidated scientific evidence at the time, which allowed for rapid legislative progress.

However, this strategy had direct consequences on implementation. To ensure feasibility and compliance with international conventions, policymakers opted to adapt pre-existing instruments, such as the rigorous and restrictive opiate control system, to regulate cannabis [2]. This choice resulted in a tendency toward strict control that created implementation challenges [6,7]. Decree 613 of 2017, which established the types of licenses, is critically analyzed for having created bottlenecks and significant delays that disproportionately affected actors with less capital, thus consolidating “normative asymmetry” [20]. This landscape was aggravated by a lack of education and public debate in the initial phases, which generated confusion between medicinal regulation and recreational legalization, and left many actors, including doctors and the farmers themselves, without the tools to navigate the new system [1]. Taken together, the analyzed scientific evidence suggests that while the law’s formulation was a political success, its practical design and subsequent implementation created the structural conditions that today define the paradox of the medical cannabis industry in Colombia.

**Figure 3:**
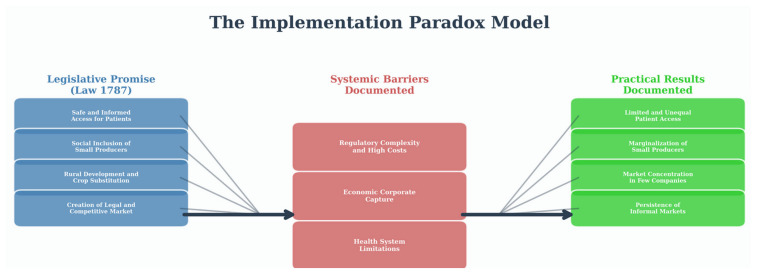
The Implementation Paradox of Medical Cannabis in Colombia: A Conceptual Synthesis. This diagram provides a clear, descriptive synthesis of the review’s findings, visually structuring the central argument.

Together, these three interconnected gaps form an implementation paradox, the conceptual model of which is presented in Figure 3.

## 4. Discussion

### 4.1. Main Findings and the Formalization of an Implementation Paradox

The findings of this scoping review are best understood through our conceptual model of the implementation paradox, which illustrates how public policy, as a social determinant of health, can produce outcomes contrary to its intent (Figure 3). This framework reveals a coherent narrative: a cutting-edge policy with noble objectives systematically undermined by structural barriers rooted in its own design. By conceptualizing the regulation as a process filtered through the experiences of key actors (patients and farmers), we analyze how its initial promise of equity has faded in practice.

#### 4.1.1. The Legislative Promise as the Horizon of Public Policy

The starting point is the legislative promise of Law 1787. The literature describes it as a milestone intended not just to create a market but to advance health and social equity [1]. Analyses of its formulation show that its approval was achieved through a skillful “strategic use of morality”, where the urgency of alleviating patient suffering legitimized a progressive reform in a complex political environment [2]. Therefore, the law was born with an explicit dual mandate: on the one hand, to guarantee access to health, and on the other, to promote social equity and rural development.

#### 4.1.2. Structural Barriers as the Central Mechanism of Inequity

The most significant finding of this review is the clarification of the mechanisms that act as a filter between policy and outcomes. The evidence is compelling that regulatory complexity, corporate market capture, and health system limitations are not isolated problems, but an interconnected system of structural barriers that function as negative social determinants of health.

On the one hand, the “normative asymmetry” described by Vélez-Torres et al. (2021) [9] details how a theoretically neutral regulatory framework is, in practice, designed to favor actors with large capital. This outcome mirrors the experience in Uruguay, where a primary focus on quality assurance led to a highly restrictive system, inadvertently limiting access and disproportionately affecting smaller local actors [6]. Furthermore, the failure to protect small producers aligns with the understanding that while social mobilization is crucial for initiating cannabis reforms, policy adoption and equitable implementation ultimately depend on the political will and the creation of elite coalitions that prioritize inclusion over corporate capture, a challenge seen across Latin America [25]. The high costs of licensing, the complexity of security protocols, and the demanding technical requirements, detailed by Riveros Santoya and Portilla Mogollón (2021) [20], function as effective entry barriers that marginalize peasant economies. On the other hand, in the health sphere, the lack of medical knowledge and training [11] and the high costs for patients [10,18] act as an almost impermeable access barrier. This implies that the effectiveness of the policy depends less on the existence of the law and more on the ability of the actors to overcome these structural obstacles.

#### 4.1.3. Practical Outcomes: The Evidence of Systemic Inequity

The final stage of the model reveals the tangible outcomes, which reflect a profound disconnect from the original promise. Instead of “safe and informed access”, the result is profound health inequity, with limited and unequal access for patients who must often resort to informal markets [18]. This dynamic, where a high-cost, limited legal supply pushes patients toward unregulated channels, is a common flaw in strictly controlled models, notably observed in Uruguay [6]. Instead of “social inclusion”, there is evidence of the marginalization and exclusion of small-scale producers, a finding corroborated by both critical qualitative studies [9] and surveys of rural actors themselves [17]. These outcomes are not unforeseen failures but the logical consequence of a system whose structural barriers systematically divert benefits away from the vulnerable populations the law intended to support. The sustained failure to mitigate these systemic barriers underscores the global finding that policy success is fundamentally dependent on addressing underlying issues of conflict, complexity, and a lack of informing evidence [8].

### 4.2. Comparison with Previous Literature and Contribution of the Study

The findings of this scoping review align with previous studies that had individually identified pieces of this paradox. For instance, the review by Castaño et al. (2025) [10] had already exhaustively synthesized the access barriers for patients, and the work of Vélez-Torres et al. (2021) [9] had offered a definitive analysis of rural exclusion. However, the unique and novel contribution of this review is the interdisciplinary synthesis of these lines of research into a unified explanatory model, the Implementation Paradox, which is validated by comparative insights from other jurisdictions. By connecting the findings from public health, critical criminology, rural sociology, and political science, we demonstrate that access barriers and the exclusion of farmers are not parallel problems, but two sides of the same coin: a systemic implementation gap. Furthermore, the model’s focus on structural barriers is corroborated by international experiences, such as Australia’s rapid but sometimes chaotic rollout, where tensions between patient demand and limited physician knowledge led to prescribing disparities, demonstrating that the challenge of translating policy into equitable practice is global [23,26]. Our Implementation Paradox Model contributes by framing this gap through the public health lens of social determinants of health, offering a unified framework for understanding how a policy’s design produces outcomes so divergent from its intentions.

Additionally, this analysis reveals a notable divergence between scientific literature and grey literature. It is striking that a large number of undergraduate theses focus almost exclusively on business plans and export potential, showing an optimistic and market-oriented vision. This approach contrasts sharply with the peer-reviewed literature analyzed here, which adopts an eminently critical stance on systemic barriers and social failures. This dichotomy suggests a gap between academic training, focused on market opportunities, and the critical analysis necessary to understand the real impact of public policies [27,28].

### 4.3. Study Limitations

Despite its methodological rigor, this scoping review has inherent limitations. First, although the search strategy was exhaustive in the selected databases, there is a risk of having omitted relevant studies not indexed or published in languages other than Spanish or English. Second, the notable heterogeneity in the methodologies of the included studies (from quantitative surveys to ethnographic analyses) makes a direct quantitative comparison and the performance of a meta-analysis difficult. Finally, the predominantly cross-sectional nature of the analyzed studies does not allow for the establishment of definitive causal relationships. While the proposed model offers strong theoretical and correlational evidence on the sequence of influence, this represents a key evidence gap.

### 4.4. Implications for Practice and Future Research

#### 4.4.1. Recommendations for Policymakers

The results offer clear implications for public policy. The central message is that to close the gap between promise and reality, interventions aimed at dismantling systemic barriers are needed:

- For the Ministry of Justice and the Ministry of Agriculture: It is crucial to review licensing requirements to create differentiated and simplified pathways for small-scale producers. This could include relaxing land tenure requirements, creating associative licensing models, and providing state technical assistance to meet quality standards.
- For the Ministry of Health and Social Protection: A national program of continuing medical education and training is required to equip health professionals with the necessary tools to prescribe with confidence. Likewise, it is essential to ensure the effective implementation of Resolution 2292 of 2021, which already updated the Health Benefits Plan (PBS) to allow for the inclusion and coverage of compounded preparations and cannabis-based medicines. The goal is to ensure that this regulation translates into real and equitable access, so that it ceases to be an economic privilege.

#### 4.4.2. Implications for Future Research

To build on these findings, a forward-looking research agenda is needed. Longitudinal studies are required to confirm the causal pathways from policy design to the production of health inequity. Furthermore, comparative research is needed, utilizing frameworks like those developed for Latin American drug reform, to identify and evaluate successful associative models that could promote equity for small-scale producers in differing political contexts [25]. Finally, pharmacoeconomic studies are essential to assess the cost-effectiveness and budgetary impact of implementing equitable access models for cannabis-based treatments within the Colombian context.

## 5. Conclusions

This scoping review has synthesized the scientific evidence on Colombia’s medical cannabis framework, demonstrating that its implementation serves as a compelling case study of how a well-intentioned policy can inadvertently function as a social determinant of health that produces inequity. The literature consistently documents that the legislative promise of a model based on social sustainability and equitable access to health has not materialized. Instead, an ecosystem has emerged marked by structural barriers that limit patient access and marginalize rural producers, favoring a model of corporate capture. The main contribution of this work is the articulation of a conceptual model that explains this gap, demonstrating that these difficulties are the result of a complex regulatory design that failed to center equity in its implementation. For Colombia to align practice with promise, future public policies must focus on dismantling these structural barriers. Only by proactively designing for health equity can the medical cannabis industry fulfill its potential to be a true engine of inclusive development and an effective tool for public health.

## Figures and Tables

**Table 1:** General Characteristics of the Included Studies.

Authors and Year	Study Type	Main Focus
Vélez-Torres et al. (2021) [9]	CE (Critical Criminology)	Analysis of the marginalization of peasants and the corporate capture of the market.
Castaño et al. (2025) [10]	NR	Identification of barriers to access to medical cannabis in Colombia.
Bustamante & Murillo (2023) [17]	CS (Survey)	Overview of medical cannabis for comprehensive rural development.
Orjuela-Rojas et al. (2021) [11]	CS (Survey)	Knowledge, beliefs, and attitudes of Colombian psychiatrists.
Rivera-Vélez (2025) [2]	PT (Process Tracing)	Strategic use of morality in the formulation of public policy.
Ledezma-Morales et al. (2020) [18]	RA	Analysis of gaps between regulations, availability, and access to products.
Cubillos Sánchez (2021) [19]	NR (Special Article)	General overview of the industry, international comparison, and therapeutic evidence.
Calderón Vallejo et al. (2017) [1]	CE (Interviews)	Interpretation of the initial regulation process and the public debate.
Riveros Santoya & Portilla M. (2021) [20]	RA	Detailed analysis of the licensing process and regulatory framework.
Maldonado Agudelo et al. (2023) [21]	CE (Descriptive)	Market and export opportunities under the legal framework.

Abbreviations: CE = Case Study; NR = Narrative Review; CS = Cross-sectional Survey; PT = Process Tracing; RA = Regulatory Analysis (or Review Article).

## Data Availability

Data are contained within the article.

## Abbreviations

The following abbreviations are used in this manuscript:

CE	Case Study
CS	Cross-sectional Survey
JBI	Joanna Briggs Institute
NR	Narrative Review
PBS	Health Benefits Plan
PCC	Population, Concept, Context
PRISMA-ScR	Preferred Reporting Items for Systematic reviews and Meta-Analyses extension for Scoping Reviews
PT	Process Tracing
RA	Regulatory Analysis
